# Remobilization and fate of sulphur in mustard

**DOI:** 10.1093/aob/mcz101

**Published:** 2019-06-10

**Authors:** Priyakshee Borpatragohain, Terry J Rose, Lei Liu, Bronwyn J Barkla, Carolyn A Raymond, Graham J King

**Affiliations:** Southern Cross Plant Science, Southern Cross University, Lismore, Australia

**Keywords:** Sulphur, sulphate, glucosinolate, storage proteins, sink, source, remobilization, assimilation, mustard, canola, brassica

## Abstract

**Background and Aims:**

Sulphur (S) is an essential macronutrient involved in numerous metabolic pathways required for plant growth. Crops of the plant family Brassicaceae require more S compared with other crops for optimum growth and yield, with most S ultimately sequestered in the mature seeds as the storage proteins cruciferin and napin, along with the unique S-rich secondary metabolite glucosinolate (GSL). It is well established that S assimilation primarily takes place in the shoots rather than roots, and that sulphate is the major form in which S is transported and stored in plants. We carried out a developmental S audit to establish the net fluxes of S in two lines of *Brassica juncea* mustard where seed GSL content differed but resulted in no yield penalty.

**Methods:**

We quantified S pools (sulphate, GSL and total S) in different organs at multiple growth stages until maturity, which also allowed us to test the hypothesis that leaf S, accumulated as a primary S sink, becomes remobilized as a secondary source to meet the requirements of GSL as the dominant seed S sink.

**Key Results:**

Maximum plant sulphate accumulation had occurred by floral initiation in both lines, at which time most of the sulphate was found in the leaves, confirming its role as the primary S sink. Up to 52 % of total sulphate accumulated by the low-GSL plants was lost through senesced leaves. In contrast, S from senescing leaves of the high-GSL line was remobilized to other tissues, with GSL accumulating in the seed from commencement of silique filling until maturity.

**Conclusion:**

We have established that leaf S compounds that accumulated as primary S sinks at early developmental stages in condiment type *B. juncea* become remobilized as a secondary S source to meet the demand for GSL as the dominant seed S sink at maturity.

## INTRODUCTION

Sulphur (S) is an essential macronutrient involved in numerous metabolic pathways required for plant growth, including the synthesis of amino acids, proteins, co-enzymes, vitamins and secondary metabolites such as glucosinolates (GSLs) and sulphoflavonoids. Plants take up S from the soil in the form of sulphate, which is then reduced to sulphide for further metabolism through S assimilation processes (Mugford *et al.*, 2010). In the S assimilation process, sulphate is activated to adenosine 5′-phosphosulphate (APS), which is the branching point for subsequent steps in which APS is reduced to form sulphide and 3′-phosphoadenosine 5′-phosphosulphate (PAPS). Sulphide is used for the synthesis of cysteine, which is the precursor for the synthesis of methionine and other primary metabolites, including storage proteins, whereas PAPS is used for sulphation of secondary metabolites, predominantly GSLs (Mugford *et al.*, 2011).

Crops of the plant family Brassicaceae, such as canola (*Brassica napus*), mustard (*Brassica juncea*), Chinese cabbage (*Brassica rapa*) and other vegetables (*Brassica oleracea*), have a larger requirement for S to achieve optimum growth and yield compared with non-Brassicaceae crops (Zhao *et al.*, 1997; Anjum *et al.*, 2012). Most S is ultimately sequestered in the mature seeds of oleiferous brassicas as the storage proteins cruciferin and napin, along with GSLs (Lakkineni and Abrol, 1992; Schatzki *et al.*, 2014). Glucosinolates have a wide range of unique properties affecting plant, animal and human health. Higher concentrations of GSLs are desired for providing the pungency to condiment mustard, while low concentrations have been selected for canola oil production.

**Fig. 1.**
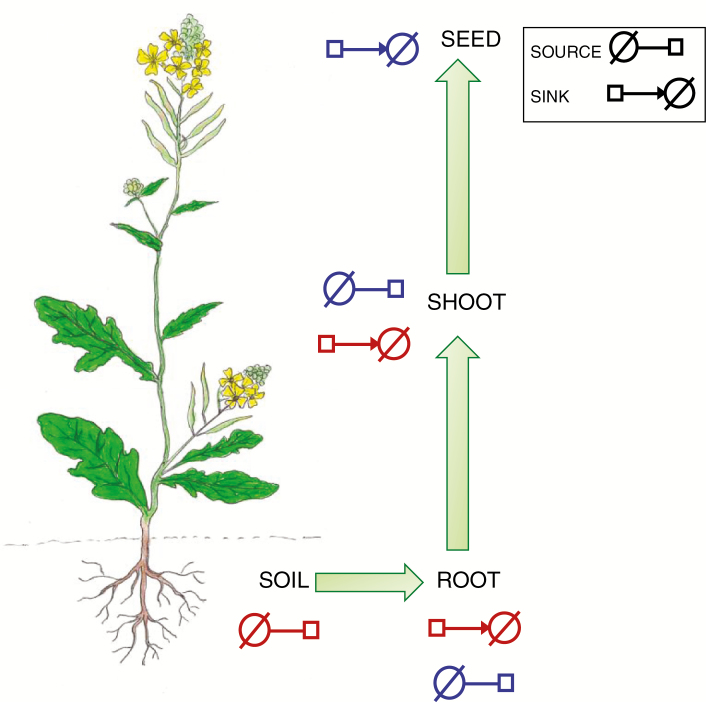
Representation of sources and sinks of S in brassicas adapted from (Borpatragohain *et al.*, 2016). Source and sink glyphs are adapted from the Systems Biology Graphical Notation (SBGN) project (http://sbgn.github.io/sbgn/). Red glyphs indicate primary S sources and sinks and blue glyphs indicate secondary S sources and sinks.

Physiological studies using the model plant *Arabidopsis thaliana* (Brassicaceae) have shown that S assimilation primarily takes place in the shoots rather than roots, and that sulphate is the major form in which S is transported and stored in plants (Hawkesford, 2000; Buchner *et al.*, 2004*b*; Hawkesford and De Kok, 2006). In this paper we refer to sources as those organs from which S and S-containing molecules are redistributed to sinks, and sinks as those organs with transient or ultimate storage of S or S-containing molecules (Fig. 1). Expression analysis of sulphate transporters identified in *A. thaliana* have confirmed their significant role in moving S between sources and sinks (Takahashi *et al.*, 2000; Yoshimoto *et al.*, 2002; Kataoka *et al.*, 2004; Cao *et al.*, 2013). The function of orthologues of these transporters also appears to be conserved to some extent in *B. oleracea* and *B. napus* (Buchner *et al.*, 2004*a*; Koralewska *et al.*, 2007; Parmar *et al.*, 2007).

In recent years, the key processes responsible for S assimilation have been extensively studied, primarily in *A. thaliana*, and reviewed in terms of precursor molecules, primary and secondary products, associated enzymes and transport systems (reviwed in Kopriva, 2006; Gigolashvili and Kopriva, 2014; Koprivova and Kopriva, 2014; Jørgensen *et al.*, 2015; Koprivova and Kopriva, 2016). Building on these findings, we developed a provisional model of the complex network of transporters, signalling molecules and transcription factors that regulate S metabolism within crop brassicas, including mustard (Borpatragohain *et al.*, 2016). We consider soil as the primary source of S and roots and shoots both as primary and secondary S sources and sinks with overlapping functions (Fig. 1). Leaves play a dual role, first as a primary sink and later in development as a secondary source. The model predicts that targeting S assimilation in crop breeding within the context of source–sink relationships could result in modified levels of seed GSL or storage proteins. At present, this approach is hindered by the lack of comprehensive data describing the uptake, distribution and fate of S and S-containing metabolites throughout crop development. Addressing this gap in *Brassica* crops would allow a better understanding of the distinct S sources and sinks (Fig. 1), and thus guide optimized agronomic practice in terms of application of S fertilizer that may affect final seed composition. We have recently established that GSLs are a key secondary S sink in seeds of a high-GSL line of Indian mustard (*B. juncea*) (Borpatragohain *et al.*, 2019). The current study was therefore undertaken to determine whether the net fluxes of S in the crop plant *B. juncea* conform to the proposed model, based on evidence primarily from *Arabidopsis*.

In order to understand the role of GSL as a potential secondary S sink we carried out a comprehensive S audit that quantified S pools (sulphate, GSL and total S) in organs of *B. juncea* at multiple growth stages until maturity, and quantified the content of GSL, sulphate and storage proteins in mature seeds. This allowed us to test the hypothesis that leaf S accumulated as a primary sink becomes remobilized as a secondary source to meet the requirements of GSL as the dominant seed S sink. The S audit was carried out in a line where 45 % of seed S is in GSL. This was compared with a line lacking GSL but where seed protein still represents a significant S sink, and where there is no yield penalty associated with lack of GSL (Borpatragohain *et al.*, 2019).

## MATERIALS AND METHODS

### Experimental design

A glasshouse trial was established at Southern Cross University, Lismore, Australia (28.8° S, 153.3° E) to carry out an S audit at five developmental stages of two *Brassica juncea* homozygous lines differing in seed GSL concentration (*B. juncea* canola-type C671 and *B. juncea* condiment-type O1493; sourced from Agriculture and Agri-Food Canada). The breeding pedigree of C671 indicates that the low-GSL trait was inherited via progenitor lines traceable to the original *B. napus* ‘Bronowski’ source (Love *et al.*, 1990; Cheng *et al.*, 2001) and carried on the A genome with extensive introgression into *B. juncea*. Fifteen pots (5 harvest stages × 3 replicates) of each of the two lines were arranged in a completely randomized design.

### Growth condition sampling strategy

Plants were grown in 150-mm-diameter, free-draining plastic pots filled with ~2 kg of potting mix comprising one-third vermiculite, one-third peat moss and one-third perlite supplemented with dolomite (1 g kg^−1^), 20 g of Osmocote^®^ Exact nutrient mix and 2 g of Micromax Premium micronutrient formula. The addition of these fertilizers provided key micro- and macronutrients in the following quantities (g per pot): 3.2 N, 1.8 P, 2.4 K, 0.5 Mg, 0.3 S, 0.4 Fe, 0.02 Ca, 0.06 Mn, 0.05 Zn, 0.03 Cu, 0.01 B and 0.005 Mo. On 6 May 2016, three seeds were sown 5 mm deep in each pot and thinned to one healthy seedling 12 d after emergence. Pots were watered to drainage daily with 0.5 L of water until harvest. Plants were harvested at five different growth stages (early vegetative, floral initiation, mid-flowering, silique filling and maturity), and tissues (leaf, stem, flower with sepal and pedicel, flower bud, green silique, silique wall, cauline leaf, green and mature seeds, roots) were separated at each harvest. Senesced leaves were collected every 3 d and kept in polypropylene bags. Each tissue sample from three biological replicates was divided into two parts: one subsample was snap-frozen in liquid nitrogen (N) and kept at −80 °C for GSL measurement and the other subsample was dried at 40 °C for 72 h for sulphate, total S and total N quantification.

Temperatures inside the glasshouse during the experiment ranged from 8.4 to 29.5 °C. Lamps (600 W HPS) were turned on for 12–16 h to initiate the flowering process at 45 d after sowing and turned off after completion of 50 % flowering.

### Measurements and chemical analysis

#### Glucosinolate

Sample preparation and extraction of GSL followed the procedure of Tian *et al.* (2005) as modified by Borpatragohain *et al.* (2019). In brief, each ground sample (~15 mg dry weight) was extracted with 1.5 mL of 70 % aqueous methanol in 2-mL Eppendorf Safe-Lock microcentrifuge tubes. To achieve a homogeneous mixture, tubes were shaken at 30 rotations s^−1^ for 30 s using a Qiagen Retsch MM 301 TissueLyser II with 5-mm stainless steel beads. The extracts were centrifuged using an angle rotor 12130 in a Sigma laboratory centrifuge at 25.155 *g* for 15 min at 7 °C. Subsequently, a 0.5-mL aliquot of each extract was transferred to a 2-mL Agilent HPLC screw-cap vial and dried under N_2_ gas. The dried samples were reconstituted in 1 mL of deionized water containing 1.17 µmol glucotropaeolin (internal standard) and sonicated for 10 min before liquid chromatography–mass spectrometry (LC–MS) analysis.

All extracts were separated using an Agilent 1290 High Performance LC–MS instrument (Agilent Technologies, Palo Alto, CA, USA) equipped with an autoinjector, binary pump, vacuum degasser and diode array detector (DAD, 1260), coupled with an Agilent 6120 quadrupole Mass Selective Detector (MSD). A Kinetex^®^ 2.6-µm EVO C18 reverse-phase column (100 × 2.1 mm internal diameter) (Phenomenex, Torrance, CA, USA) was used, with temperature set at 30 °C. A linear gradient elution programme was applied, comprising a mobile phase containing Milli-Q water with 0.01 % trifluoroacetic acid (TFA) (solvent A) and acetonitrile with 0.005 % TFA (solvent B) at a flow rate of 0.3 mL min^−1^ and 5 µL injection volume. The 18-min run consisted of 0 % B (0–8 min), 25 % B (10 min), 100 % B (13 min) and 0 % B (14–18 min). The MSD was operated in atmospheric pressure ionization-electrospray mode with the following parameters: fragmenter, 150; capillary voltage, 3000 V (negative); drying gas flow, 12.0 L min^−1^ (N_2_); vaporizer temperature, 350 °C; nebulizer pressure, 35 psi; drying gas temperature, 350 °C. Absorbance was monitored at 210, 280 and 360 nm. Single-ion monitoring mode was set to detect seven ions simultaneously in negative-ion mode using four available mass-selective detection signal channels: signal 1, sinigrin at *m*/*z* ratio 358 for 0–8 min and glucotropaeolin at *m*/*z* ratio 408 for 8–18 min; signal 2, progoitrin and epiprogoitrin at *m*/*z* ratio 388 for 0–18 min; signal 3, glucoiberin at *m*/*z* ratio 422 for 0–10 min and gluconasturtiin at *m*/*z* ratio 422 for 10–18 min; signal 4, gluconapin at *m*/*z* ratio372 for 0–18 min. Glucotropaeolin, not found in brassicas, was used as the internal standard to monitor the performance of MS (Fahey *et al.*, 2001). All LC–MS settings and parameters were optimized based on the manufacturer’s recommendations and a number of flow injection experiments.

All the organic solvents used in the analysis were HPLC or LCMS grade. Commercial GSL standards were obtained from PhytoLab, Germany. Although we measured six different types of GSL in each sample, only the major compounds sinigrin and gluconapin were summed for total GSL calculation.

#### Sulphate

The concentration of sulphate was measured by ion chromatography (883 Basic Ion Chromatography plus, Metrohm, Australia) following the modified method of Frankowski (2016). Ground samples were extracted using Milli-Q water (1 g water to 40 g oven-dried tissue) by shaking on a horizontal shaker for 1 h followed by centrifugation at 5.292 *g* with a swinging-bucket rotor 11150 for 10 min at 20 °C using a Sigma 4K15 table-top centrifuge. Subsequently, 5 mL of supernatant of each extract was filtered through a 0.45-µm hydrophilic syringe filter (Sartorius, Germany) before analysis by ion chromatography. Sulphate anions were separated using a Metrosep A Supp 4–250/4.0 column (Metrohm, Australia) with a 17-min run of an isocratic elution system consisting of 1.7 mmol L^−1^ sodium bicarbonate (NaHCO_3_) and 1.8 mmol L^−1^ sodium carbonate (Na_2_CO_3_) at 1 mL min^−1^ flow rate. For quantification of sulphate, Sigma–Aldrich Multi Anion Standard 1 was used as a standard.

#### Total sulphur and protein

Total S concentrations in the tissue were determined in a 0.2-g subsample using a CS combustion analyser model SC832 (LECO, MI, USA) at the Environmental Analysis Laboratory, Southern Cross University, Australia. To prevent the loss of volatile GSL because of oven-drying and grinding prior to total S measurement, we used green and mature intact seeds and freeze-dried, ground materials of other plant tissues.

To measure total S in the protein fractions of mature seeds, samples were prepared by trichloroacetic acid (TCA) precipitation of proteins (Barkla *et al.*, 2016). Seeds (~50 mg dry weight) were ground in 1.5 mL of absolute cold methanol with a 5-mm tungsten carbide bead in 2-mL Eppendorf Safe-Lock microcentrifuge tubes using a Qiagen Retsch MM 301 TissueLyser II at 15 rotations s^−1^ for 2 min. Supernatant was discarded after centrifugation at 25.155 *g* for 20 min at 4 °C and residual methanol was evaporated using an Eppendorf Concentrator 5301 for 1 h at room temperature. Dried samples were reconstituted in 1 mL of deionized water and mixed with 200 µL of 10× concentrated TE buffer (final: 10 mm Tris/HCl, pH 7.6; 1 mm EDTA, pH 8) and 200 µL of 0.3 % (w/v) sodium deoxycholate. Samples were then precipitated sequentially by 72 % (w/v) TCA, followed by 90 % methanol. Supernatant was discarded after centrifugation at 21.912 *g* for 20 min at 4 °C and washing with methanol was repeated twice. Samples were dried under N_2_ gas before measuring total S by CS analyser as above.

In addition, we measured the total protein concentration in the mature seeds by the Bradford method (Bradford, 1976) using bovine serum albumin (BSA) as the standard. For the Bradford assay, ground seed samples (10 mg) were dissolved in Milli-Q water (600 µL) and shaken at 15 rotations s^−1^ for 15 s using a Qiagen Retsch MM 301 TissueLyser II followed by 1 h of sonication in dry ice prior to protein estimation to achieve a homogeneous mixture, and measured according to Barkla *et al.* (2016). Absorbance was measured at 595 nm using a BMG Labtech CLARIOstar microplate reader. The concentration of protein in the sample was calculated by blank-corrected linear regression fitting of the BSA standards.

Glucosinolate, sulphate, protein and total S accumulated per organ were calculated by multiplying the concentration by the respective dry weight of the organ. We calculated the percentage of total S, represented as GSL-S, by dividing the molecular weights of the two S atoms found in GSL molecules by the total molecular weight of GSL. Likewise, the proportion of total S, represented as sulphate-S, was calculated based on the percentage molecular weight. The other possible forms of S were termed residual-S and calculated by subtracting GSL-S and sulphate-S from total S in that particular tissue (Supplementary Data Table S1).

### Statistical analyses

Data analysis was undertaken using Genstat 64-bit release 18.1 (VSN International) software. For each mustard line the following sets of analyses were run. (1) Two-way unbalanced ANOVA was run as a general linear model (GLM) fitting plant part, development stage and their interaction. Treatment means were predicted from the GLM together with pairwise least significant differences (LSDs) (*P* = 0.05). A Bonferroni test (*P* = 0.05) was used to test differences between plant parts at each development stage (Supplementary Data Table S2). (2) For the factors of development stage and plant part, one-way ANOVAs were run for S, GSL, sulphate and protein, and LSDs (*P* = 0.05) were calculated to test differences between paired sets of means (Supplementary Data Tables S3 and S4).

## RESULTS

### Phenology, biomass production and biomass partitioning between organs

**Fig. 2.**
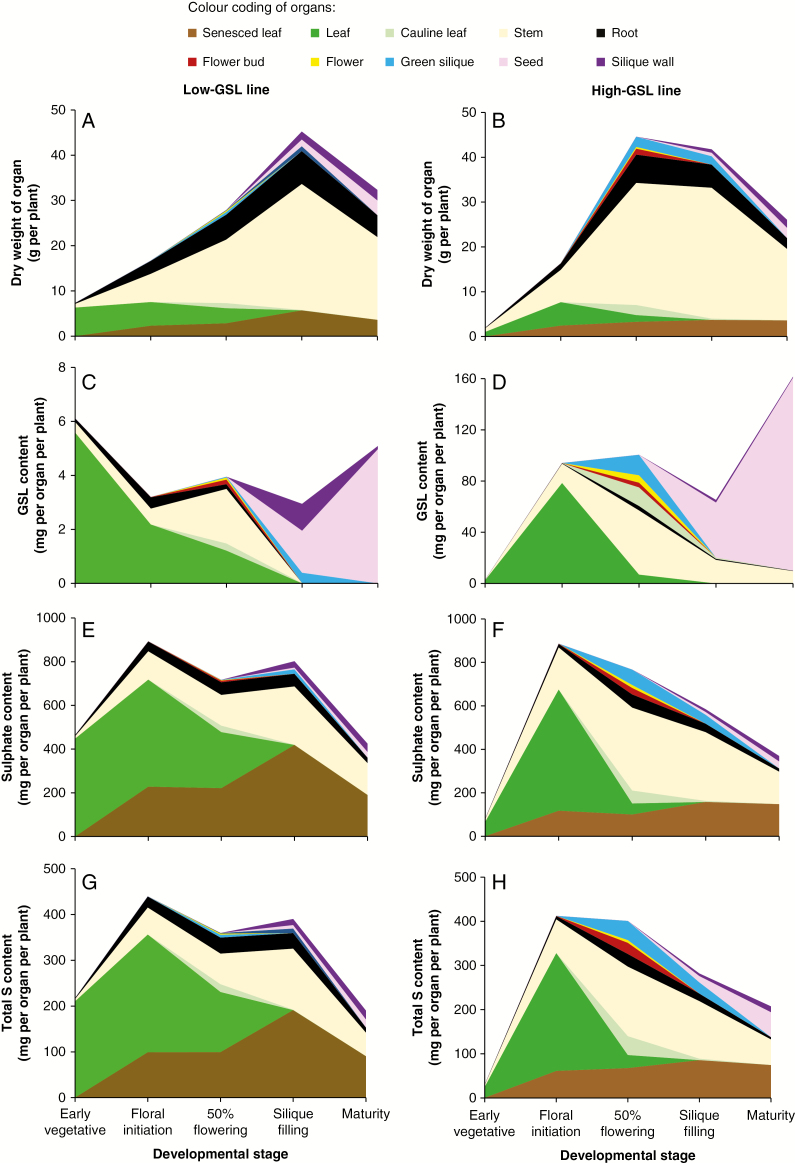
Accumulation of biomass, GSL, sulphate and total S in low- and high-GSL mustard lines at five developmental stages. Details are given in Supplementary Data Tables S2 and S4.

Phenology and growth patterns differed between the low- and the high-GSL line. The date of bolting was on average 20 d earlier for plants of the high-GSL line than for the low-GSL line. Although the high-GSL line had a shorter growth period prior to 50 % flowering (45 compared with 62 d after sowing), it accumulated more biomass by this stage (44.6 versus 27.8 g per plant) (Supplementary Data Tables S3 and S4). Whilst the high-GSL line attained peak biomass accumulation by 50 % flowering, in the low-GSL line biomass accumulation continued to increase until the silique filling stage (Fig. 2A, B; Supplementary Data Table S4).

The mean seed yield of the low-GSL line was 3.3 g per plant with a harvest index (ratio of seed biomass to above-ground biomass) of 0.14, compared with 2.3 g per plant and a harvest index of 0.11 in the high-GSL line (Supplementary Data Table S3).

### Glucosinolate, sulphate and S accumulation during plant development

**Fig. 3.**
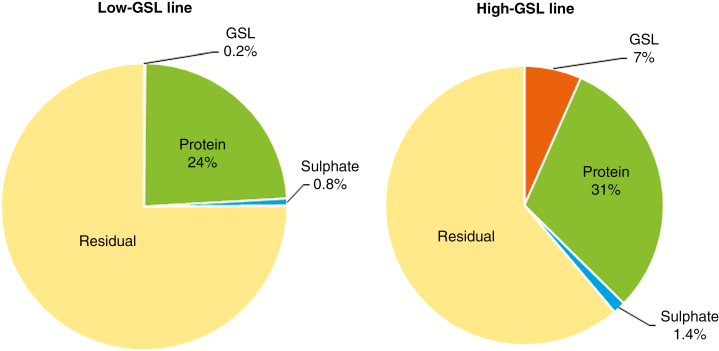
Mass distribution of S-containing seed components (GSL, protein, sulphate and residual) in the low- and high-GSL lines of *B. juncea* at maturity. ‘Residual’ indicates oil and other seed components. Details are given in Supplementary Data Tables S2 and S5.

The accumulation of GSL in the low-GSL canola type line was negligible (<6.5 mg per plant) throughout the entire plant growth period (Fig. 2C; Supplementary Data Table S4) compared with the 162 mg of GSL per plant accumulated in the high-GSL condiment type line by maturity (Fig. 2C, D; Supplementary Data Table S4). Approximately half of the latter had been accumulated by floral initiation, predominately located in the leaves (Fig. 2D; Supplementary Data Tables S2a and S4). When rosette leaves started to abscise at flowering, cauline leaves and the reproductive organs of flower bud, flower and green silique contained >40 % of total GSLs in the high-GSL plants. However, by silique filling around half of the total plant GSL was located in the seeds, with the remainder in the stem, green silique and silique wall or pericarp. By maturity ~93 % of total plant GSL was located in seeds (Fig. 2D; Supplementary Data Table S3). Seed GSLs accounted for 7 % of the total seed mass of the high-GSL line, compared with 0.2 % of the seed mass in the low-GSL line (Fig. 3; Supplementary Data Tables S3 and S5). In the seeds of the low-GSL line protein accounted for 24 % of the seed mass, compared with 30 % in the high-GSL line (Fig. 3; Supplementary Data Tables S3 and S5).

Plants of both the high- and low-GSL lines accumulated a maximum of ~890 mg of sulphate per plant (Fig. 2E, F; Supplementary Data Table S2). Maximum plant sulphate accumulation had occurred by floral initiation in both lines, at which time most of the sulphate was found in the leaves, which confirms their role as primary sink. Interestingly, up to 52.3 % of total sulphate accumulated by the low-GSL plants was lost through senesced leaves, which was not the case for the high-GSL line. Green seeds at the silique filling stage accounted for about half of the sulphate stored in mature seeds of both lines. At maturity, sulphate accounted for 1.4 % of the total seed mass of the high-GSL line, compared with 0.8 % in the low-GSL line (Fig. 3; Supplementary Data Table S3). By maturity, 6–8 % of total plant sulphate was located within the seed for both lines (Supplementary Data Table S3).

The accumulation and partitioning of total plant S mirrored that of sulphate in both the high- and low-GSL lines (Fig. 2G, H; Supplementary Data Table S2). The low-GSL line had larger leaves and a longer vegetative phase, which increases the opportunity to accumulate more S (Fig. 2G; Supplementary Data Table S2). This was also reflected in increased biomass of the low-GSL line during the vegetative stage (Fig. 2A; Supplementary Data Table S4). Notably, while senesced leaves contained negligible amounts of GSL (below the detection limit of LC–MS), they contained around 50 and 36 % of total plant S at maturity in the low- and high-GSL lines, respectively (Fig. 2C, D, G, H; Supplementary Data Table S3). Total S accounted for 0.5 and 2.5 % of total seed mass at maturity for low- and high-GSL plants, respectively (Fig. 3; Supplementary Data Table S3).

### Distribution and forms of S over time

**Fig. 4.**
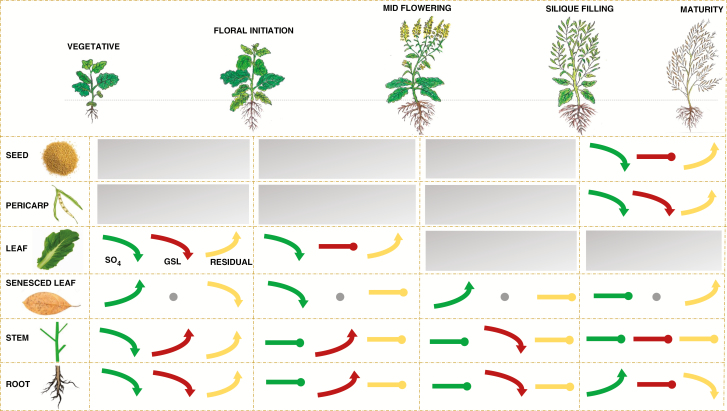
Net fluxes of S between source and sink tissues of the high-GSL *B. juncea* line based on experimental analysis of sulphate-S, GSL-S and residual-S during development_._ Green arrows indicate sulphate-S, red arrows indicate GSL-S and yellow arrows indicate residual-S. Upward arrows indicate accumulation or an increase in content, downward arrows indicate translocation or decrease, and a straight line with a dot indicates stasis or storage. Grey dots indicate undetected S forms. Grey shaded boxes indicate stages at which organ was not present. Details are given in Supplementary Data Table S1.

The total S content of the high-GSL plants increased significantly from the early vegetative stage to flower initiation and then remained stable until maturity. The plants of the low-GSL line followed a similar trend, except that a small but significant (*P* < 0.05) decline in total S content was observed between flower initiation and maturity (Supplementary Data Table S4). At the whole-plant level there were significant differences in the distribution of sulphate-S between the high- and low-GSL lines. Sulphate was the major form of S in the early vegetative stage of the high-GSL line, and then decreased until maturity. In contrast, in the low-GSL line sulphate remained the predominant form of S throughout development (Supplementary Data Table S1). The proportion of total S localized in GSL (GSL-S) in the high-GSL line increased with the age of plants to a maximum of 13 % at maturity, whereas GSL-S was negligible (0.1–0.5 %) in the low-GSL line throughout development. At maturity, 45 % of total S in the seeds of the high-GSL line was GSL-S_,_ compared with only 5 % of total S in the low-GSL line as GSL-S (Fig. 4; Supplementary Data Table S1).

### Theoretical mass balance calculation of the fate of S in the seed secondary sink

To calculate the theoretical stoichiometric ratio of GSL-S and S in protein (protein-S) components contributing to seed mass (Table 1A), we used a GSL value of 100 µmol g^−1^ of seed mass as reported for mustard (*B. juncea*) (Bisht *et al.*, 2009). Seed storage protein was estimated at 30 % of seed mass (Nesi *et al.*, 2008). The ratio of cruciferin to napin (1:0.3) was estimated from the available literature for *Brassica* species (Crouch and Sussex, 1981). This indicates that the proportion of total S stored in the seed as GSL-S is twice that of protein-S (Table 1A).

**Table 1.** (A) Theoretical mass balance calculation of the fate of S in seeds, accounting for 95 % of S

Sink molecule	Molecular weight (g mol^−1^)	Number of S atoms per molecule	S per molecule (%)	Molecule (mg g^−1^ seed)	S in sink molecule as % of seed mass
GSL (gluconapin)	373.39	2	16	37.3^1^	**0.64**
Cruciferin	400 000^2^	9^3^	0.07	230^4^	0.02
Napin	9000^2^	13^3^	5	66^4^	0.31
Total storage protein					**0.33**

^1^The concentration of GSL found in the seeds of mustard (*B. juncea*) is ~100 µmol (sinigrin or gluconapin) per gram of seed mass (Bisht *et al.*, 2009).

^2^Molecular weights of cruciferin and napin were obtained from the UniProt database and available literature (Perera *et al.*, 2016).

^3^Number of S atoms present in cruciferin and napin was calculated from the amino acid sequences of the respective proteins from UniProt database, on the basis of one S atom for each S-containing amino acid (methionine and cysteine).

^4^Seed storage proteins typically represent up to 30 % of *Brassica* seed mass (Nesi *et al.*, 2008; Dimov *et al.*, 2012), where 60 % of the total protein is represented by globulin-like cruciferins and 20 % by 2S albumin-class napins (Crouch and Sussex, 1981) and the remainder by oleosins and other proteins.

### Experimental evidence of the fate of S in mature seeds of low- and high-GSL lines

**Fig. 5.**
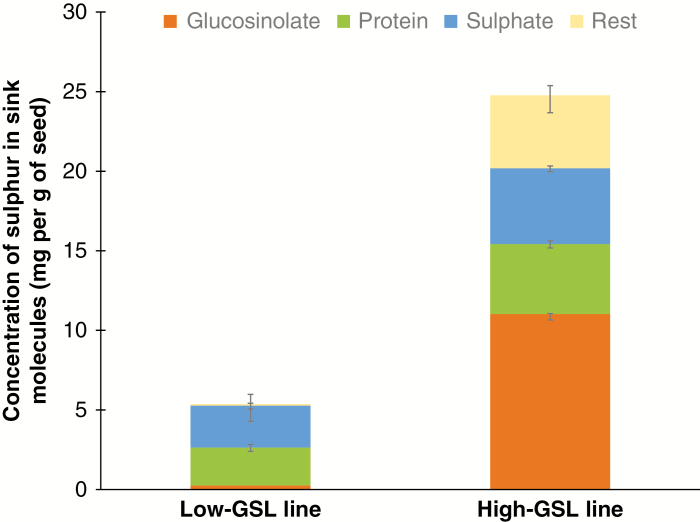
Concentration of S in different seed S fractions (GSL-S, sulphate-S, protein-S and residual-S) of low- and high-GSL lines. Details are given in Supplementary Data Table S5.

Mature seeds of the high GSL line contained 25 mg of S per gram of seeds (Fig. 5; Supplementary Data Table S5). This line accumulated 210 µmol of GSL per gram of seeds, of which 99.3 % was sinigrin (Supplementary Data Table S6). In contrast, seeds of the low-GSL line had 5 mg of total S and only 0.6 µmol of GSL per gram of seeds (Fig. 5; Supplementary Data Table S2). Sulphur stored in the seed protein (protein-S) of the high-GSL line was twice that of the low-GSL line (Table 1B; Fig. 5; Supplementary Data Table S5).

**Table:** Table 1. (B) Experimental evidence of fate of S in mature seeds of low- and high-GSL lines. Details are given in Supplementary Data Tables S2 and S5

Sink molecule	Molecular weight (g mol^−1^)	Number of S atom per molecule	S per molecule (%)	High GSL		Low GSL	
				Molecule (mg g^−1^ seed)	S in sink molecule as % of seed mass	Molecule (mg g^−1^ seed)	S in sink molecule as % of seed mass
GSL (sinigrin)	397.46	2	16	66.3	**1.10**	1.52	**0.03**
Total protein				307.4	**0.44**	239.5	**0.24**
Total S				24.77	2.48	5.37	0.54

## DISCUSSION

Internal transport systems for sulphate and GSLs are well described in the context of sources and sinks for the model plant *Arabidopsis* (reviewed in Gigolashvili and Kopriva, 2014; Borpatragohain *et al.*, 2016), with only a few studies in canola using radiolabelled S supply to detect total S and sulphate remobilization (Balint and Rengel, 2011). However, a comprehensive understanding of the uptake, distribution and fate of S and S-containing metabolites such as GSL is lacking for brassica field crops, including Indian mustard. We considered soil as the primary source of S and roots and shoots both as primary and secondary S sources and sinks with overlapping functions (Fig. 1).

There appeared to be adequate total S supply to both lines, given that the total S uptake was similar (around 400 mg per plant). Thus, the 25× lower GSL content in the low-GSL line compared with the high-GSL line appears likely due to a disruption in the GSL biosynthetic pathways or GSL transport. This is consistent with evidence that reduction in activity or specificity of methylthioalkylmalate synthase genes (Liu *et al.*, 2011) and the transcription factor MYB 28 (Augustine *et al.*, 2013) in other *Brassica* species has been shown to confer low seed GSL concentrations. Moreover, the breeding pedigree of this low-GSL line indicates that the low-GSL trait was inherited via progenitor lines traceable to the original *B. napus* ‘Bronowski’ source (Love *et al.*, 1990; Cheng *et al.*, 2001) and carried on the A genome with extensive introgression into *B. juncea*.

The uptake and allocation of S to different S pools in the low-GSL line was similar to that observed in commercially grown double-low oilseed rape (canola) lines. GSLs accounted for <0.5 % of the total S pool in leaves of the low-GSL plants, similar to canola, where GSLs account for <1 % of the total S present in leaves (Blake-Kalff *et al.*, 1998). Indeed, the GSL concentration in the seeds of the low-GSL line (0.6 µmol g^−1^) was lower than the mean GSL concentrations of 18–57 µmol g^−1^ in field-grown canola crops (Sarda *et al.*, 2014). This lack of seed-sink strength for S in the low-GSL line was reflected in the low remobilization of S from senescing leaves to other tissues compared with the high-GSL line (Fig. 2G, H). This suggests that there was minimal translocation of sulphate from senescing leaves in the low-GSL line, indicative of adequate S availability. This is consistent with evidence from canola, where substantial remobilization of sulphate from senescing leaves only occurs under conditions of S deficiency (Dubousset *et al.*, 2009; Abdallah *et al.*, 2010, 2011; Balint and Rengel, 2011; Sarda *et al.*, 2014; Maillard *et al.*, 2015). In terms of ensuring S supply to seeds in a GSL-independent manner, we have previously shown in an S dose–response study of the low-GSL line that the total seed S and seed yield reached a plateau with excess S supply (Borpatragohain *et al.*, 2019). The reason for the significant loss of total S from mid-flowering to maturity in the low-GSL line is not known, but similar observations have been made in field-grown canola plants (Karamanos *et al.*, 2007; Karamanos, 2013).

In contrast to the low-GSL line, in the high-GSL line both GSL and sulphate appeared to be remobilized from senescing leaves to other tissues. Leaf sulphate concentrations declined significantly from >100 mg g^−1^ at floral initiation to around 33 mg g^−1^ by mid-flowering, and were only 30–50 mg g^−1^ in senesced leaf tissue, supporting their role as secondary S sources. Leaf GSL concentrations declined significantly from 15 mg g^−1^ at floral initiation to 5 mg g^−1^ at mid-flowering, with no detectable concentrations of GSLs in senesced leaf (Supplementary Data Table S2). Given that the period during which the GSL concentration in the leaves declined (as they began to senesce) overlapped with the early stages of GSL accumulation in developing seeds (Fig. 2D; Supplementary Data Table S2), it is possible that GSL molecules were transported intact from senescing leaves to seeds either directly or via transient sink tissues such as the stem or pericarp tissue. Indeed, earlier studies in other *Brassica* species have found that this phenomenon occurs in conjunction with the expression of GSL transporter genes (GTRs) in plant tissues, leading the authors to conclude that the GSLs were transported as intact molecules (Petersen *et al.*, 2002; Brown *et al.*, 2003; Nour-Eldin *et al.*, 2012; Andersen and Halkier, 2014). However, to the best of our knowledge there is currently no direct evidence for the transport of intact GSL molecules in any *Brassica* species, and while our data would support such a phenomenon, there are several other possibilities that could explain the loss of GSL from senescing leaves. It is quite plausible that GSL molecules were first broken down into sulphate, which was then translocated either to other transient S sink organs (stem or pericarp tissue) or the developing seeds either to be stored as sulphate or re-synthesized into GSLs. It is also possible that during senescence some GSLs come into contact with the myrosinase enzyme due to a breakdown of cellular or organ compartments, to produce volatile isothiocyanates, with the associated S lost to the atmosphere. However, given that GSLs accounted for <5 % of total leaf S at any growth stage, while sulphate accounted for ~ 60–90 % of leaf S, the majority of S was clearly re-partitioned within the plant. This also supports the view of leaves as the site of S metabolite synthesis (Gigolashvili and Kopriva, 2014). Significantly larger concentrations of GSLs were observed in the green seeds during silique filling compared with other plant parts throughout development, with the GSL concentration increasing in the seeds until maturity (Supplementary Data Table S2). Our previously developed model (Borpatragohain *et al.*, 2016) would suggest that either developing seeds are capable of *de novo* synthesis of GSLs or that synthesized GSLs are transported from the source organs (cauline leaf, silique wall or pericarp) to developing seeds.

Within the glasshouse environment the concentration of seed GSL (66 mg g^−1^) was consistent with concentrations typically achieved under field conditions (Ramchiary *et al.*, 2007; Rout *et al.*, 2015). The protein-S and GSL-S concentrations in seed of the high-GSL line were consistent with our theoretical mass balance calculation (Table 1A), where, crucially, the low-molecular-weight napins have 10-fold more S atoms per molecule than GSLs.

One of the primary motivations for this S audit study was to determine whether the net fluxes of S in the crop plant *B. juncea* conform to the proposed source–sink model based on evidence primarily from *Arabidopsis* (Fig. 4 of Borpatragohain *et al.*, 2016). The patterns and major components of S accumulation and remobilization throughout *B. juncea* development (Fig. 2) appear consistent with this model (Fig. 1). We have established that leaf S components that accumulated as primary S sinks at early developmental stages in condiment-type *B. juncea* become remobilized as a secondary S source to meet the demand for GSL as the dominant seed S sink at maturity. This is analogous to findings in monocot crops such as wheat, where S is remobilized from flag leaves to the grain (protein) S sink (Monaghan *et al*., 1999). Based on studies in canola (Abdallah *et al.*, 2010, 2011), there may be an interaction between N availability and S dynamics. Future studies can now build on the current study to examine N × S interactions in *B. juncea*. Our evidence for S remobilization from leaves as primary S sink suggests that up- or downregulation of signalling molecules that mediate between secondary S sinks and sources may help modulate economically valuable S compounds in brassica seed.

## SUPPLEMENTARY DATA

Supplementary data are available online at https://academic.oup.com/aob and consist of the following. Table S1: percentage distribution of total S in GSL-S, sulphate-S and residual-S fractions in plant tissues over five developmental stages in low- and high-GSL mustard lines. Table S2a: accumulation (mg per organ per plant) of GSL, inorganic sulphate, total S and total protein in various plant parts of low- and high-GSL *B. juncea* lines at five developmental stages. Table S2b: concentration of GSL, inorganic sulphate, total S and total protein in various plant parts of low- and high-GSL *B. juncea* lines at five developmental stages. Table S3a: concentration, accumulation and distribution of biomass, GSL, sulphate, protein and total S among plant tissues in the low-GSL line for each development stage. Table S3b: concentration, accumulation and distribution of biomass, GSL, sulphate, protein and total S among plant tissues in the high-GSL line for each development stage. Table S4: accumulation of biomass, total S, GSL, sulphate and total protein in low- and high- GSL mustard lines at five crop development stages. Table S5: concentration of seed S sinks and concentration of S in sink molecules in low- and high-GSL lines of *B. juncea*. Table S6: concentration of individual GSLs in the seeds of high and low-GSL lines.

mcz101_suppl_Supplementary_Table_S1

mcz101_suppl_Supplementary_Table_S2

mcz101_suppl_Supplementary_Table_S3

mcz101_suppl_Supplementary_Table_S4

mcz101_suppl_Supplementary_Table_S5

mcz101_suppl_Supplementary_Table_S6

## FUNDING

P.B. received scholarship funding from Australian Mustard Oil Pty. Ltd, Australia, DBT-AAU Center of Assam Agricultural University, India, and Southern Cross Plant Science Centre, Southern Cross University, Australia. The authors declare that although they have a financial relationship with Australian Mustard Oil Pty. Ltd, the latter had no influence whatsoever on the conduct, materials used, or outcome of this research.
