# Supplementary material for: Remobilization and fate of sulphur in mustard
Source: Ann Bot. 2019 Jun 10;124(3):471–80. doi: 10.1093/aob/mcz101 (PMC6798836; doi:10.1093/aob/mcz101)
Supplement: mcz101_suppl_Supplementary_Table_S1 [file mcz101_suppl_supplementary_table_s1.docx]

## **Supplementary Table 1.**

Percent distribution of total Sulphur (S) in GSL-S, sulphate-S and residual-S fractions in plant tissues over five developmental stages in low- and high-GSL mustard lines. We considered 16 percent of GSL molecule as GSL-S which represented 2 molecules of S found on sinigrin or gluconapin molecule. Likewise, 33% of sulphate molecule as sulphate-S which represented 1 atom of S found as inorganic sulphate molecule. The percentage of S in other possible forms was termed as residual S and was calculated by subtracting the combined S in GSL and sulphate from the total S in that particular tissue.

| Sulphur fractions | Plant Part | Low-GSL line | | | | | High-GSL line | | | | |
| --- | --- | --- | --- | --- | --- | --- | --- | --- | --- | --- | --- |
|  |  | Early vegetative | Floral initiation | 50% flowering | Silique filling | Maturity | Early vegetative | Floral initiation | 50% flowering | Silique filling | Maturity |
| % GSL-S | Leaf | 0.44 | 0.14 | 0.15 |  |  | 1.74 | 4.88 | 3.90 |  |  |
|  | Cauline leaf |  |  | 0.26 | 0.18 |  |  |  | 5.68 | 4.60 |  |
|  | Root | 1.13 | 0.29 | 0.08 | 0.00 | 0.00 | 2.18 | 0.93 | 2.01 | 0.84 | 0.82 |
|  | Stem | 1.27 | 0.17 | 0.50 | 0.00 | 0.00 | 0.91 | 3.29 | 5.29 | 2.34 | 2.77 |
|  | Flower bud |  | 0.28 | 0.46 |  |  |  | 3.06 | 2.41 |  |  |
|  | Flower |  |  | 0.46 |  |  |  |  | 15.57 |  |  |
|  | Green silique |  |  | 0.56 | 0.66 |  |  |  | 6.12 | 9.55 |  |
|  | Silique wall |  |  |  | 1.24 | 0.10 |  |  |  | 5.63 | 0.93 |
|  | seed |  |  |  | 3.42 | 4.71 |  |  |  | 50.94 | 44.44 |
|  | Senesced leaf | 0.00 | 0.00 | 0.00 | 0.00 | 0.00 | 0.00 | 0.00 | 0.00 | 0.00 | 0.00 |
|  | Whole plant | 0.47 | 0.12 | 0.18 | 0.13 | 0.44 | 1.59 | 3.79 | 4.17 | 4.71 | 12.95 |
| % sulphate-S | Leaf | 70.90 | 63.52 | 65.05 |  |  | 89.86 | 69.59 | 57.38 |  |  |
|  | Cauline leaf |  |  | 57.82 | 16.44 |  |  |  | 46.64 | 50.4 |  |
|  | Root | 82.32 | 62.14 | 54.75 | 56.46 | 75.78 | 92.04 | 74.3 | 71.28 | 75.54 | 95.25 |
|  | Stem | 73.57 | 73.23 | 70.08 | 66.77 | 93.98 | 92.68 | 84.57 | 80.52 | 80.85 | 85.95 |
|  | Flower bud |  | 36.78 | 43.39 |  |  |  | 41.43 | 39.23 |  |  |
|  | Flower |  |  | 26.22 |  |  |  |  | 71.96 |  |  |
|  | Green silique |  |  | 40.34 | 68.05 |  |  |  | 54.41 | 54.22 |  |
|  | Silique wall |  |  |  | 72.72 | 66.55 |  |  |  | 71.45 | 62.73 |
|  | seed |  |  |  | 35.84 | 49.19 |  |  |  | 30.22 | 19.20 |
|  | Senesced leaf | 16.98 | 76.56 | 74.11 | 73.04 | 69.81 | 35.14 | 64.05 | 49.21 | 61.34 | 65.88 |
|  | Whole plant | 70.99 | 67.65 | 66.41 | 68.44 | 74.48 | 89.15 | 71.56 | 63.72 | 69.11 | 59.32 |
| % residual-S | Leaf | 28.66 | 36.34 | 34.80 | 83.38 |  | 8.40 | 25.53 | 38.72 |  |  |
|  | Cauline leaf |  |  | 41.92 |  |  |  |  | 47.68 | 45.00 |  |
|  | Root | 16.54 | 37.57 | 45.16 | 43.54 | 24.22 | 5.78 | 24.77 | 26.71 | 23.63 | 3.93 |
|  | Stem | 25.16 | 26.60 | 29.41 | 33.23 | 6.02 | 6.42 | 12.14 | 14.2 | 16.81 | 11.27 |
|  | Flower bud |  | 62.94 | 56.15 |  |  |  | 55.51 | 58.36 |  |  |
|  | Flower |  |  | 73.33 |  |  |  |  | 12.47 |  |  |
|  | Green silique |  |  | 59.11 | 31.30 |  |  |  | 39.46 | 36.23 |  |
|  | Silique wall |  |  |  | 26.03 | 33.35 |  |  |  | 22.92 | 36.34 |
|  | seed |  |  |  | 60.74 | 46.1 |  |  |  | 18.84 | 36.36 |
|  | Senesced leaf | 83.02 | 23.44 | 25.89 | 26.96 | 30.19 | 64.86 | 13.71 | 12.37 | 9.79 | 11.22 |
|  | Whole plant | 28.54 | 32.22 | 33.41 | 31.44 | 25.07 | 9.26 | 21.35 | 25.62 | 17.41 | 19.49 |
