# Supplementary material for: Remobilization and fate of sulphur in mustard
Source: Ann Bot. 2019 Jun 10;124(3):471–80. doi: 10.1093/aob/mcz101 (PMC6798836; doi:10.1093/aob/mcz101)
Supplement: mcz101_suppl_Supplementary_Table_S2 [file mcz101_suppl_supplementary_table_s2.docx]

## **Supplementary Table 2 a.**

Accumulation (mg per organ per plant) of glucosinolate (GSL), inorganic sulphate, total sulphur and total protein in various plant parts of low and high-GSL *B. juncea* lines at five developmental stages. Values are predicted means from a General Linear Model fitted for each trait within each line. Means followed by the same letter within each column are not significantly different based on calculated least significant differences (LSD) in a Bonferroni test (p=0.05). Total nitrogen concentrations from a LECO CNS analyser were converted to protein concentrations by multiplying with the conversion factor of 6.25 (Jones, 1941).

| Developmental stages | Plant part | GSL | | | Sulphate | | | Total sulphur | | Total protein | | |
| --- | --- | --- | --- | --- | --- | --- | --- | --- | --- | --- | --- | --- |
|  |  | Low | High | Low | | High | Low | | High | | Low | High |
| LSD (p=0.05) |  | 1.42 | 16.05 | 72.23 | | 84.20 | 30.26 | | 47.91 | | 636 | 486 |
| Early vegetative | Leaf | 5.59 c | 2.56 a | 447.9 d | | 65.99 ab | 210.49 g | | 24.47 a | | 5009 f | 925 abc |
|  | Root | 0.13 a | 0.10 a | 4.84 a | | 2.17 a | 1.96 a | | 0.79 a | | 38 a | 22 a |
|  | Stem | 0.39 a | 0.25 a | 11.35 a | | 12.65 a | 5.14 ab | | 4.55 a | | 179 a | 191 a |
|  | Senesced leaf | 0.00 | 0.00 | 0.14 a | | 0.70 a | 0.28 a | | 0.66 a | | 3 a | 17 a |
| Floral initiation | Leaf | 2.19 ab | 78.47 d | 490.26 d | | 558.05 e | 257.13 g | | 267.13 d | | 3699 e | 3614 d |
|  | Root | 0.42 a | 0.41 a | 45.14 a | | 16.51 a | 24.20 ab | | 7.40 a | | 357 ab | 123 a |
|  | Stem | 0.59 a | 15.06 ab | 129.2 abc | | 193.03 bc | 58.8 abcd | | 76.04 abc | | 571 abc | 834 ab |
|  | Flower bud | 0.01 a | 0.14 a | 0.54 a | | 0.93 a | 0.48 a | | 0.75 a | | 19 a | 17 a |
|  | Senesced leaf | 0.00 | 0.00 | 228.26 c | | 117.62 ab | 99.32 de | | 61.17 ab | | 537 abc | 962 abc |
| 50% flowering | Leaf | 1.22 a | 6.92 a | 256.52 c | | 50.88 ab | 131.37 ef | | 29.54 a | | 1585 bcd | 551 a |
|  | Cauline leaf | 0.27 a | 14.51 ab | 29.70 a | | 59.48 ab | 17.11 ab | | 42.48 ab | | 314 ab | 581 a |
|  | Root | 0.17 a | 3.46 a | 57.73 ab | | 61.21 ab | 35.12 abc | | 28.61 a | | 662 abc | 492 a |
|  | Stem | 2.02 a | 50.14 cd | 140.2 abc | | 381.12 de | 66.67 bcd | | 157.68 c | | 1260abcd | 1860 c |
|  | Flower bud | 0.17 a | 3.66 a | 7.87 a | | 29.73 ab | 6.04 ab | | 25.25 a | | 167 a | 474 a |
|  | Flower | 0.08 a | 5.85 a | 2.23 a | | 13.49 a | 2.83 a | | 6.25 a | | 70 a | 109 a |
|  | Green silique | 0.03 a | 15.95 ab | 1.10 a | | 70.72 ab | 0.91 a | | 43.30 ab | | 33 a | 693 a |
|  | Senesced leaf | 0.00 | 0.00 | 221.50 c | | 100.15 ab | 99.56 de | | 67.79 abc | | 627 abc | 791 a |
| Silique filling | Cauline leaf | 0.01 a | 0.95 a | 0.51 a | | 5.19 a | 1.03 a | | 3.43 a | | 6 a | 58 a |
|  | Root | 0.00 | 0.86 a | 58.02 ab | | 38.82 ab | 34.23 abc | | 17.12 a | | 606 abc | 293 a |
|  | Stem | 0.00 | 18.35 abc | 267.01 c | | 316.20 cd | 133.22 ef | | 130.28 bc | | 2485 de | 1789 bc |
|  | Silique wall | 0.99 a | 2.02 a | 29.01 a | | 12.77 a | 13.29 ab | | 5.95 a | | 187 a | 88.4 a |
|  | Green seed | 1.56 a | 43.08 bc | 8.16 a | | 12.75 a | 7.58 ab | | 14.06 a | | 468 abc | 265 a |
|  | Green silique | 0.39 a | 14.56 ab | 19.92 a | | 41.26 ab | 9.75 ab | | 25.35 a | | 202 a | 397 a |
|  | Senesced leaf | 0.00 | 0.00 | 419.47 d | | 157.7 abc | 191.31 fg | | 85.64 abc | | 1237abcd | 895 abc |
| Maturity | Root | 0.00 | 0.26 a | 25.28 a | | 14.93 a | 11.11 ab | | 5.22 a | | 321 ab | 101 a |
|  | Stem | 0.00 | 9.65 a | 145 abc | | 149 abc | 51.6 abcd | | 57.89 ab | | 1199abcd | 786 a |
|  | Silique wall | 0.11 a | 0.74 a | 38.10 a | | 24.77 ab | 19.07 ab | | 13.16 a | | 317 ab | 180 a |
|  | seed | 4.97 bc | 150.99 e | 25.92 a | | 32.55 ab | 17.55 ab | | 56.47 ab | | 783 abc | 700 a |
|  | Senesced leaf | 0.00 | 0.00 | 190 bc | | 148 abc | 90.80 cde | | 74.72 abc | | 1715 cd | 911abc |

## **Supplementary Table 2 b.**

Concentration (mg per g of organ) of glucosinolate (GSL), inorganic sulphate, total sulphur and total protein in various plant parts of low and high-GSL *B. juncea* lines at five developmental stages. Values are predicted means from a General Linear Model fitted for each trait within each line. Means followed by the same letter within each column are not significantly different based on calculated least significant differences (LSD) in a Bonferroni test (p=0.05). Total nitrogen concentrations from a LECO CNS analyser were converted to protein concentrations by multiplying with the conversion factor of 6.25 (Jones, 1941).

| Developmental stages | Plant part | GSL | | Sulphate | | Total sulphur | | Total protein | |
| --- | --- | --- | --- | --- | --- | --- | --- | --- | --- |
|  |  | Low | High | Low | High | Low | High | Low | High |
| LSD (p=0.05) |  | 0.47 | 2.83 | 15.68 | 10.37 | 5.46 | 4.81 | 70 | 47 |
| Early vegetative | Leaf | 0.86 abc | 2.45 ab | 71.03 cd | 65.45 g | 33.37 ef | 24.26 h | 794 i | 917 m |
|  | Root | 1.40 abc | 0.90 a | 22.00 ab | 15.00abcd | 8.90 abc | 5.43 abc | 175abcdefg | 151 bcd |
|  | Stem | 0.58 ab | 0.29 a | 13.89 a | 15.70abcd | 6.29 abc | 5.66 abc | 219 bcdefg | 238 defg |
|  | Senesced leaf | 0.00 a | 0.00 a | 8.71 a | 13.60abcd | 17.10 cd | 12.9bcdef | 179abcdefg | 332 ghij |
| Floral initiation | Leaf | 0.40 ab | 15.04 c | 93.74 d | 106.60 h | 49.17 g | 51.03 i | 707 i | 690 l |
|  | Root | 0.13 ab | 0.31 a | 15.68 a | 11.3 abc | 8.41 abc | 5.08 abc | 124 abcde | 85 ab |
|  | Stem | 0.10 ab | 2.07 ab | 20.75 ab | 26.70abcde | 9.44 abc | 10.5abcde | 91 abcd | 115 abc |
|  | Flower bud | 0.21 ab | 3.84 ab | 14.85 a | 29.80bcdef | 13.45abc | 23.93 h | 532 h | 542 k |
|  | Senesced leaf | 0.00 a | 0.00 a | 98.60 d | 48.20 fg | 42.90 fg | 25.07 h | 232cdefg | 394 j |
| 50% flowering | Leaf | 0.36 ab | 5.21 ab | 76.82 cd | 33.61 def | 39.34 fg | 19.51efgh | 475 h | 364 ij |
|  | Cauline leaf | 0.23 ab | 6.73 b | 26.48 ab | 26.50abcde | 15.3 bcd | 18.9 efgh | 279 fg | 258 efgh |
|  | Root | 0.04 a | 0.58 a | 10.51 a | 9.70 abc | 6.39 abc | 4.54 abc | 120 abcde | 78 ab |
|  | Stem | 0.20 ab | 1.76 ab | 9.99 a | 14.00abcd | 4.75 ab | 5.79 abc | 90 abc | 68 ab |
|  | Flower bud | 0.27 ab | 2.88 ab | 12.66 a | 22.00abcde | 9.72 abc | 19 efgh | 269 fg | 358 ij |
|  | Flower | 0.27 ab | 14.93 c | 7.48 a | 34.83 def | 9.51 abc | 16 defgh | 236 defg | 282efghi |
|  | Green silique | 0.28 ab | 6.64 b | 10.07 a | 31.00 cdef | 8.32 abc | 19 efgh | 298 g | 301 fghij |
|  | Senesced leaf | 0.00 a | 0.00 | 77.64 cd | 30.00bcdef | 34.90 ef | 20.60 fgh | 220 bcdefg | 240 efgh |
| Silique filling | Cauline leaf | 0.14 ab | 4.43 ab | 6.98 a | 17.80 abcd | 14.1 bcd | 11.8abcdef | 78 ab | 198 cde |
|  | Root | 0.00 a | 0.17 a | 7.95 a | 7.48 a | 4.69 ab | 3.30 ab | 83 ab | 56 ab |
|  | Stem | 0.00 a | 0.63 a | 9.58 a | 10.80 abcd | 4.78 abc | 4.46 abc | 89 abc | 61 ab |
|  | Silique wall | 0.44 ab | 2.62 ab | 16.67 a | 17.00 abcd | 7.63 abc | 7.81 abcd | 108 abcde | 116 abc |
|  | Green seed | 1.02 bc | 55.37 d | 5.44 a | 16.00 abcd | 5.06 ab | 18.10 efgh | 313 g | 341 hij |
|  | Green silique | 0.36 ab | 7.36 b | 19.24 a | 22.30abcde | 9.42 abc | 13.7 cdefg | 194abcdefg | 214 def |
|  | Senesced leaf | 0.00 a | 0.00 | 73.45 cd | 42.51 ef | 33.50 ef | 23.08 gh | 217 bcdefg | 241 defg |
| Maturity | Root | 0.00 a | 0.10 a | 5.22 a | 6.14 a abcd | 2.29 a | 2.15 a | 66 a | 42 a |
|  | Stem | 0.00 a | 0.65 a | 7.96 a | 9.37 ab | 2.82 a | 3.63 ab | 66 a | 49 a |
|  | Silique wall | 0.15 a | 0.43 a | 16.49 a | 14.00abcd | 8.25 abc | 7.42 abcd | 137 abcdef | 102 ab |
|  | seed | 1.52 c | 66.33 e | 7.93 a | 14.28 abcd | 5.37 ab | 24.77 gh | 239 efg | 307 fghij |
|  | Senesced leaf | 0.00 a | 0.00 a | 52.46 bc | 40.74 ef | 25.03 de | 20.60 fgh | 473 h | 251 efgh |
