# Supplementary material for: Remobilization and fate of sulphur in mustard
Source: Ann Bot. 2019 Jun 10;124(3):471–80. doi: 10.1093/aob/mcz101 (PMC6798836; doi:10.1093/aob/mcz101)
Supplement: mcz101_suppl_Supplementary_Table_S3 [file mcz101_suppl_supplementary_table_s3.docx]

## **Supplementary Table 3 a.**

Concentration, accumulation and distribution of biomass, GSL, sulphate, protein and total S, among plant tissues in the low-GSL line for each development stage. Means within a column that are followed by a common letter for a given development stage are not significantly different at p=0.05.

| Developmental stages | Plant Part | Biomass | | GSL | | | sulphate | | | sulphur | | | Protein | | |
| --- | --- | --- | --- | --- | --- | --- | --- | --- | --- | --- | --- | --- | --- | --- | --- |
|  |  | accumulation  (g organ^-1^) | % of plant total | Conc. (mg g^-1^) | Content  (mg organ^-1^) | % of plant total | Conc. (mg g^-1^) | Content  (mg organ^-1^) | % of plant total | Conc. (mg g^-1^) | Content  (mg organ^-1^) | % of plant total | Conc. (mg g^-1^) | Content  (mg organ^-1^) | % of plant total |
| Early vegetative | Leaf | 6.3 b | 86 | 0.9 a | 5.6 b | 91 | 71.0 c | 448 b | 96 | 33.4 c | 211 b | 97 | 794 b | 5010 b | 96 |
|  | Root | 0.2 a | 3 | 1.4 a | 0.1 a | 2 | 22.0 b | 4.8 a | 1 | 8.9 a | 2.0 a | 0.9 | 174 a | 39 a | 0.7 |
|  | Stem | 0.8 a | 11 | 0.6 a | 0.4 a | 6 | 13.9 ab | 11.3 a | 2 | 6.3 a | 5.1 a | 2 | 219 a | 179 a | 3 |
|  | Senesced leaf | 0.02 a | 0.2 | 0.0 | 0.0 | 0.00 | 8.7 a | 0.14 a | 0.03 | 17.1 b | 0.28 a | 0.13 | 180 a | 2.9 a | 0.06 |
| Total plant^-1^ |  | 7.36 |  | 2.84 | 6.12 |  | 115.63 | 464.29 |  | 65.66 | 217.86 |  | 1367.58 | 5230.04 |  |
| LSD (5%) |  | 1.89 |  | 1.46 | 3.31 |  | 8.87 | 93.17 |  | 3.97 | 44.37 |  | 92.61 | 1732.32 |  |
| Floral Initiation | Leaf | 5.2 c | 31 | 0.4 b | 2.2 b | 68 | 93.7 b | 491 d | 55 | 49.2 c | 257 d | 59 | 707 d | 3699 c | 71 |
|  | Root | 2.9 b | 17 | 0.1 a | 0.4 a | 13 | 15.7 a | 45.1 ab | 5 | 8.4 a | 24.2 a | 6 | 124 a | 357 ab | 7 |
|  | Stem | 6.2 c | 37 | 0.1 a | 0.6 a | 18 | 20.8 a | 129.2 bc | 14 | 9.4 a | 58.8 b | 13 | 91.7 a | 571 b | 11 |
|  | Flower bud | 0.04 a | 0.22 | 0.2 ab | 0.01 a | 0.26 | 14.9 a | 0.54 a | 0.06 | 13.5 a | 0.48 a | 0.11 | 532 c | 19.2 a | 0.37 |
|  | Senesced leaf | 2.3 b | 14 | 0.0 | 0.00 | 0.00 | 98.6 b | 228 c | 26 | 42.9 b | 99.3 c | 23 | 232 b | 537 b | 10 |
| Total plant^-1^ |  | 16.7 |  | 0.8 | 3.2 |  | 243 | 893 |  | 123 | 440 |  | 1687 | 5183 |  |
| LSD (5%) |  | 1.2 |  | 0.2 | 1.3 |  | 26.6 | 104 |  | 5.6 | 30.1 |  | 102 | 453 |  |
| 50% flowering | Leaf | 3.3 c | 12 | 0.4 a | 1.2 ab | 31 | 76.8 b | 256.5 c | 36 | 39.3 c | 131 c | 37 | 475 e | 1585 e | 34 |
|  | Cauline leaf | 1.1 ab | 4 | 0.2 bc | 0.3 a | 7 | 26.5 a | 29.7 s | 4 | 15.3 b | 17.1 a | 5 | 280 cd | 314 ab | 7 |
|  | Root | 5.5 d | 20 | 0.04 a | 0.2 a | 4 | 10.5 a | 57.7 a | 8 | 6.4 ab | 35.1 ab | 10 | 120 a | 662 bc | 14 |
|  | Stem | 14.0 e | 50 | 0.2 ab | 2.02 b | 51 | 10.0 a | 140.2 b | 20 | 4.8 a | 66.7 b | 19 | 90 a | 1260 d | 27 |
|  | Flower bud | 0.6 a | 2 | 0.3 cd | 0.2 a | 4 | 12.6 a | 7.9 a | 1 | 9.7 ab | 6.0 a | 2 | 269 cd | 167 a | 4 |
|  | Flower | 0.3 a | 1 | 0.3 cd | 0.08 a | 2 | 7.5 a | 2.2 a | 0.3 | 9.5 ab | 2.8 a | 0.8 | 236 bc | 70 a | 1.4 |
|  | Green silique | 0.1 a | 0.4 | 0.3 cd | 0.03 a | 0.7 | 10.1 a | 1.1 a | 0.15 | 8.3 ab | 0.9 a | 0.25 | 298 d | 33 a | 0.7 |
|  | Senesced leaf | 2.9 bc | 10 | 0.00 | 0.00 | 0.00 | 77.6 b | 221.5 c | 31 | 34.9 c | 99.6 c | 28 | 220 b | 627 bc | 13 |
| Total plant-1 |  | 27.9 |  | 1.65 | 3.95 |  | 231 | 717 |  | 128 | 359.61 |  | 1987 | 4717.84 |  |
| LSD (5%) |  | 1.9 |  | 0.11 | 1.35 |  | 19.9 | 75.4 |  | 8.3 | 33.46 |  | 44.5 | 325.22 |  |
| Silique filling | Cauline leaf | 0.07 a | 0.2 | 0.14 ab | 0.01 a | 0.4 | 7.0 a | 0.5 a | 0.06 | 14.1 c | 1.03 a | 0.26 | 78.6 a | 5.7 a | 0.1 |
|  | Root | 7.3 b | 16 | 0.0 | 0.0 | 0 | 8.0 a | 58 a | 7 | 4.7 a | 34.2 b | 9 | 83.1 a | 606.3 c | 12 |
|  | Stem | 27.9 c | 62 | 0.0 | 0.0 | 0 | 10.0 ab | 267 b | 33 | 4.8 a | 133 c | 34 | 89.2 a | 2485 e | 48 |
|  | Silique wall | 1.7 a | 4 | 0.4 b | 0.9 ab | 34 | 16.7 bc | 29 a | 4 | 7.6 ab | 13.3 ab | 3 | 107.7 a | 187 ab | 4 |
|  | Green seed | 1.5 a | 3 | 1.02 c | 1.6 b | 53 | 5.4 a | 8.2 a | 1 | 5.06 a | 7.6 ab | 2 | 312.7 c | 469 bc | 9 |
|  | Green silique | 1.04 a | 2 | 0.4 ab | 0.4 ab | 13 | 19.2 c | 19.9 a | 3 | 9.4 b | 9.8 ab | 3 | 194.9 b | 202 ab | 4 |
|  | Senesced leaf | 5.7 b | 13 | 0.0 | 0.0 | 0 | 73.5 d | 419 c | 52 | 33.5 d | 191 d | 49 | 216.7 b | 1237 d | 24 |
| Total plant^-1^ |  | 45.2 |  | 2.0 | 3.0 |  | 139.3 | 802 |  | 79.2 | 390 |  | 1082.9 | 5192.62 |  |
| LSD (5%) |  | 2.2 |  | 0.39 | 1.16 |  | 7.95 | 56.18 |  | 2.75 | 26.90 |  | 81.80 | 307.05 |  |
| Maturity | Root | 4.9 b | 15 | 0.0 | 0.0 | 0.0 | 5.2 a | 25.3 a | 6 | 2.3 a | 11.1 a | 6 | 66.3 a | 321 a | 8 |
|  | Stem | 18.3 c | 57 | 0.0 | 0.0 | 0.0 | 8.0 a | 145.5 b | 34 | 2.8 a | 51.6 b | 27 | 65.6 a | 1199 c | 28 |
|  | Silique wall | 2.3 a | 7 | 0.15 a | 0.11 a | 2.2 | 16.5 a | 38.1 a | 9 | 8.3 a | 19.1 ab | 10 | 137.4 a | 317 a | 7 |
|  | seed | 3.3 a | 10 | 1.52 c | 5.0 c | 97.8 | 7.9 a | 25.9 a | 6 | 5.4 a | 17.6 ab | 9 | 239.5 b | 783 b | 18 |
|  | Senesced leaf | 3.6 ab | 11 | 0.0 | 0.0 | 0.0 | 52.5 b | 190 b | 45 | 25 b | 90.8 c | 48 | 472.9 c | 1715 d | 40 |
| Total plant^-1^ |  | 31.3 |  | 1.67 | 5.09 |  | 90 | 425 |  | 43.8 | 190 |  | 982 | 4335 |  |
| LSD (5%) |  | 1.4 |  | 0.222 | 0.958 |  | 14.2 | 73.2 |  | 6.27 | 33.6 |  | 71.59 | 357 |  |
| Harvest Index (%) |  | 13.7 |  |  | 97.79 |  |  | 12.4 |  |  | 19.91 |  |  | 34.04 |  |

## **Supplementary Table 3 b.**

Concentration, accumulation and distribution of biomass, GSL, sulphate, protein and total S, among plant tissues in the high-GSL line for each development stage. Means within a column that are followed by a common letter for a given development stage are not significantly different at p=0.05.

| Developmental stages | Plant Part | Biomass | | GSL | | | sulphate | | | sulphur | | | Protein | | |
| --- | --- | --- | --- | --- | --- | --- | --- | --- | --- | --- | --- | --- | --- | --- | --- |
|  |  | accumulation  (g organ^-1^) | % of plant total | Conc. (mg g^-1^) | Content  (mg organ^-1^) | % of plant total | Conc. (mg g^-1^) | Content  (mg organ^-1^) | % of plant total | Conc. (mg g^-1^) | Content  (mg organ^-1^) | % of plant total | Conc. (mg g^-1^) | Content  (mg organ^-1^) | % of plant total |
| Early vegetative | Leaf | 1.01 b | 50 | 2.5 b | 2.6 b | 88 | 65.5 b | 65.9 b | 81 | 24.3 c | 24.5 b | 80 | 917.7 d | 925 b | 80 |
|  | Root | 0.14 ab | 7 | 0.9 a | 0.1 a | 4 | 15.0 a | 2.2 a | 3 | 5.4 a | 0.8 a | 3 | 150.9 a | 21.8 a | 2 |
|  | Stem | 0.8 ab | 40 | 0.3 a | 0.3 a | 9 | 15.7 a | 12.7 a | 16 | 5.7 a | 4.6 a | 15 | 238.1 b | 191 s | 17 |
|  | Senesced leaf | 0.05 a | 3 | 0.0 | 0.0 | 0 | 13.6 a | 0.7 a | 0.8 | 12.9 b | 0.7 a | 2 | 332 c | 16.9 a | 1.5 |
| Total plant^-1^ |  | 2.01 |  | 3.6 | 2.9 |  | 109.8 | 81.5 |  | 48.3 | 30.5 |  | 1639 | 1155 |  |
| LSD (5%) |  | 0.88 |  | 1.44 | 1.31 |  | 7.64 | 22.81 |  | 2.09 | 8.93 |  | 42.14 | 274 |  |
| Floral Initiation | Leaf | 5.2 c | 32 | 15.0 d | 78.5 c | 84 | 107 d | 558 c | 63 | 51.0 d | 267 c | 65 | 690 d | 3614 d | 65 |
|  | Root | 1.5 ab | 9 | 0.3 a | 0.4 a | 0.4 | 11.3 a | 16.5 a | 2 | 5.1 a | 7.4 a | 2 | 84.6 a | 123 ab | 2 |
|  | Stem | 7.2 d | 44 | 2.1 ab | 15.1 ab | 16 | 26.7 b | 193 b | 22 | 10.5 b | 76.0 b | 18 | 115.4 a | 833 bc | 15 |
|  | Flower bud | 0.03 a | 0.19 | 3.8 bc | 0.1 a | 0.15 | 29.8 b | 0.9 a | 0.1 | 23.9 c | 0.8 a | 0.2 | 541.7 c | 17.0 a | 0.3 |
|  | Senesced leaf | 2.4 b | 15 | 0.0 | 0.0 | 0.0 | 48.2 c | 118 ab | 13 | 25.1 c | 61.1 ab | 15 | 394 b | 962 c | 17 |
| Total plant^-1^ |  | 16.4 |  | 21.3 | 94.1 |  | 223 | 886 |  | 115.7 | 412 |  | 1826 | 5549 |  |
| LSD (5%) |  | 1.67 |  | 2.37 | 7.62 |  | 9.96 | 118.7 |  | 4.51 | 26.97 |  | 80.69 | 718 |  |
| 50% flowering | Leaf | 1.5 ab | 3 | 5.2 bc | 6.9 a | 7 | 33.6 cd | 50.9 a | 7 | 19.5 b | 29.5 a | 7 | 364 c | 551 a | 10 |
|  | Cauline leaf | 2.2 ab | 5 | 6.7 c | 14.5 a | 14 | 26.5 cd | 59.5 a | 8 | 18.9 b | 42.5 a | 10 | 259 b | 581 a | 10 |
|  | Root | 6.3 b | 14 | 0.6 a | 3.5 a | 3 | 9.7 a | 61.2 a | 8 | 4.5 a | 28.6 a | 7 | 78 a | 491 a | 9 |
|  | Stem | 27.2 c | 61 | 1.8 ab | 50.1 b | 50 | 14.0 ab | 381 b | 50 | 5.8 a | 157.7 b | 39 | 68.3 a | 1860 b | 34 |
|  | Flower bud | 1.3 ab | 3 | 2.9 abc | 3.7 a | 4 | 22.4 bc | 29.7 a | 4 | 19. b | 25.3 a | 6 | 358 c | 475 a | 9 |
|  | Flower | 0.4 a | 0.9 | 14.9 d | 5.9 a | 6 | 34.8 d | 13.5 a | 2 | 16.1 b | 6.3 a | 2 | 281 b | 109 a | 2 |
|  | Green silique | 2.3 ab | 5 | 6.6 c | 16.0 a | 16 | 30.7 cd | 70.7 a | 9 | 18.8 b | 43.3 a | 11 | 301 bc | 693 a | 12 |
|  | Senesced leaf | 3.3 ab | 7 | 0.0 | 0.0 | 0.0 | 30.4 cd | 100.1 a | 13 | 20.6 b | 67.8 a | 17 | 240 b | 791 a | 14 |
| Total plant-1 |  | 44.6 |  | 38.7 | 100.5 |  | 202 | 767 |  | 123.3 | 401 |  | 1949 | 5551 |  |
| LSD (5%) |  | 4.64 |  | 3.79 | 20.69 |  | 10.47 | 121.9 |  | 6.34 | 77.01 |  | 64.15 | 650.20 |  |
| Silique filling | Cauline leaf | 0.3 a | 0.7 | 4.4 bc | 1.0 a | 1 | 17.9 ab | 5.2 a | 0.9 | 11.8 bc | 3.4 a | 1 | 199 c | 57.8 a | 2.0 |
|  | Root | 5.2 c | 12 | 0.2 a | 0.9 a | 1 | 7.5 a | 38.8 a | 7 | 3.3 a | 17.1 a | 6 | 56.5 a | 293 a | 8 |
|  | Stem | 29.2 d | 70 | 0.6 a | 18.4 b | 23 | 10.8 ab | 316 c | 54 | 4.5 a | 130.3 c | 46 | 61.3 a | 1789 c | 47 |
|  | Silique wall | 0.8 ab | 2 | 2.6 ab | 2.0 a | 3 | 16.8 ab | 12.8 a | 2 | 7.8 ab | 6.0 a | 2 | 116 b | 88.4 a | 2 |
|  | Green seed | 0.8 ab | 2 | 55.4 e | 43.1 c | 54 | 16.4 ab | 12.8 a | 2 | 18.1 d | 14.1 a | 5 | 341 f | 265 a | 7 |
|  | Green silique | 1.9 ab | 4 | 7.4 c | 14.6 ab | 18 | 22.3 b | 41.3 a | 7 | 13.7 cd | 25.4 a | 9 | 214 d | 396 a | 10 |
|  | Senesced leaf | 3.7 bc | 9 | 0.0 | 0.0 | 0 | 42.5 c | 158 b | 27 | 23.1 e | 85.6 b | 30 | 241 e | 895 b | 24 |
| Total plant^-1^ |  | 41.8 |  | 70.6 | 79.8 |  | 134 | 585 |  | 82.3 | 282 |  | 1229 | 3785 |  |
| LSD (5%) |  | 3.11 |  | 3.47 | 12.91 |  | 12.63 | 67.95 |  | 4.98 | 34.21 |  | 9.53 | 468.21 |  |
| Maturity | Root | 2.43 ab | 9 | 0.1 a | 0.3 a | 0.2 | 6.1 a | 14.9 a | 4 | 2.2 a | 5.2 a | 3 | 41.6 a | 101 a | 10 |
|  | Stem | 15.9 c | 61 | 0.7 a | 9.7 a | 6 | 9.4 a | 149.4 b | 40 | 3.6 a | 57.9 b | 28 | 49.3 a | 786 b | 78 |
|  | Silique wall | 1.8 a | 7 | 0.4 a | 0.7 a | 0.5 | 14.0 a | 24.8 a | 7 | 7.4 a | 13.2 a | 6 | 102 b | 180 a | 1 |
|  | seed | 2.3 ab | 9 | 66.3 c | 151 c | 93 | 14.3 a | 32.6 a | 9 | 24.8 b | 56.5 b | 27 | 307 d | 701 b | 3 |
|  | Senesced leaf | 3.6 b | 14 | 0.0 | 0.0 | 0.0 | 40.7 b | 148 b | 40 | 20.6 b | 74.7 b | 36 | 251 c | 912 b | 7 |
| Total plant^-1^ |  | 26.0 |  | 67.5 | 162 |  | 84.5 | 369 |  | 58.6 | 207 |  | 751 | 2680 |  |
| LSD (5%) |  | 1.41 |  | 2.67 | 24.02 |  | 13.98 | 56.69 |  | 5.87 | 23.95 |  | 24.39 | 209.74 |  |
| Harvest Index (%) |  | 11.41 |  |  | 93.6 |  |  | 15.8 |  |  | 35.6 |  |  | 42.0 |  |
