# Supplementary material for: Remobilization and fate of sulphur in mustard
Source: Ann Bot. 2019 Jun 10;124(3):471–80. doi: 10.1093/aob/mcz101 (PMC6798836; doi:10.1093/aob/mcz101)
Supplement: mcz101_suppl_Supplementary_Table_S4 [file mcz101_suppl_supplementary_table_s4.docx]

## **Supplementary Table 4.**

Accumulation of biomass, total S, GSL, sulphate and total protein in low- and high GSL mustard lines at five crop development stages. Means within a column that are followed by a common letter are not significantly different at p =0.05.

| Developmental stages | Biomass (g per plant) | | Total S (mg per plant) | | GSL (mg per plant) | | Sulphate (mg per plant) | | Total protein (mg per plant) | |
| --- | --- | --- | --- | --- | --- | --- | --- | --- | --- | --- |
|  | Low | High | Low | High | Low | High | Low | High | Low | High |
| LSD (P=0.05) | 4.9 | 11.5 | 103.6 | 162.9 | 3.9 | 41.5 | 219.5 | 271.5 | 1828.1 | 1741.2 |
| Early vegetative | 7.4 a | 2.0 a | 217.9 a | 30.5 a | 6.1 a | 2.9 a | 464.3 a | 81.5 a | 5230.1 a | 115.4 a |
| Floral initiation | 16.7 b | 16.4 b | 439.9 b | 412.5 bc | 3.2 a | 94.1 b | 893.4 b | 886.2 b | 5183.3 a | 5548.9 c |
| 50% flowering | 27.9 c | 44.6 c | 359.6 b | 400.9 bc | 4.0 a | 100.5 b | 716.9 b | 766.8 b | 4717.8 a | 5550.9 c |
| Silique filling | 45.2 d | 41.8 c | 390.4 b | 281.8 b | 3.0 a | 79.8 b | 802.1 b | 584.7 b | 5192.6 a | 3784.7 bc |
| Maturity | 31.3 c | 26.1 b | 190.1 a | 207.5 b | 5.1 a | 161.6 c | 425.1 a | 369.4 b | 4335.7 a | 2680.2 b |
