# Supplementary material for: Remobilization and fate of sulphur in mustard
Source: Ann Bot. 2019 Jun 10;124(3):471–80. doi: 10.1093/aob/mcz101 (PMC6798836; doi:10.1093/aob/mcz101)
Supplement: mcz101_suppl_Supplementary_Table_S5 [file mcz101_suppl_supplementary_table_s5.docx]

## **Supplementary Table 5:**

Concentration of seed S sinks and concentration of S in sink molecules (mg per g of seed) in low- and high-GSL lines of *B. juncea*. Means are shown + standard deviation. For calculating GSL-S, we assumed 16 % of a GSL molecule was GSL-S, which represented two molecules of S found on the C_3_ side-chain aliphatic GSL-sinigrin and C_4_ side-chain aliphatic GSL-gluconapin molecules. Likewise, for calculating sulphate-S, we assumed 33 % of sulphate molecule was sulphate-S. This represented 1 molecule of S found in inorganic sulphate. We measured protein-S (mg per g of seeds) by trichloroacetic acid (TCA) precipitation (method section 2.3 c). The residual-S fraction was calculated by subtracting the combined S in GSL-S, sulphate-S and protein-S from the total seed S concentration.

| Seed S sink | Concentration of seed S sinks (mg per g of seed) + SD | | Concentration of S in sink molecules (mg per g of seed) +SD | | Sulphur as % of total seed Sulphur | |
| --- | --- | --- | --- | --- | --- | --- |
|  | Low-GSL line | High-GSL line | Low-GSL line | High-GSL line | Low-GSL line | High-GSL line |
| Glucosinolate | 1.52+0.18 | 66.33+2.22 | 0.25+0.03 | 11.03+0.37 | 4.71 | 44.51 |
| Total protein | 239+6.00 | 307+4.27 | 2.38+0.20 | 4.40+0.24 | 44.30 | 17.76 |
| Sulphate | 7.93+0.44 | 14.28+0.54 | 2.64+0.15 | 4.76+0.21 | 49.19 | 19.20 |
| Residual |  |  | 0.10+0.60 | 4.59+1.09 | 1.80 | 18.52 |
| Total sulphur | 5.37+0.36 | 24.77+0.69 | 5.37+0.36 | 24.77+0.69 |  |  |
