# Supplementary material for: Remobilization and fate of sulphur in mustard
Source: Ann Bot. 2019 Jun 10;124(3):471–80. doi: 10.1093/aob/mcz101 (PMC6798836; doi:10.1093/aob/mcz101)
Supplement: mcz101_suppl_Supplementary_Table_S6 [file mcz101_suppl_supplementary_table_s6.docx]

## **Supplementary Table 6:**

Concentration of individual GSLs (µmol g^-1^) in the seeds of high and low-GSL lines. Means are shown + standard deviation.

| Genotypes | Sinigrin | Progoitrin | Epiprogoitrin | Glucoiberin | Gluconasturtiin | Gluconapin |
| --- | --- | --- | --- | --- | --- | --- |
| Low-GSL line | 0.12 ± 0.3 | 0.07 ± 1.1 | 0.01+0.2 | Not detected | Not detected | 0.42± 0.33 |
| High-GSL line | 209 ± 0.1 | 0.01 ± 0.04 | Not detected | 0.23 ± 0.03 | 0.1 ± 0.02 | 0.99 ± 0.01 |
